# Vascular Endothelial Growth Factor and Placental Growth Factor in Conjunction with Vascular Endothelial Growth Factor Receptor-1 May Exert Dual Effects Within the Kidney and Brain in Patients with Type 2 Diabetes Mellitus and Normoalbuminuric Diabetic Kidney Disease

**DOI:** 10.3390/ijms27093752

**Published:** 2026-04-23

**Authors:** Ligia Petrica, Florica Gadalean, Adrian Vlad, Daliborca Vlad, Victor Dumitrascu, Tutac Paul, Flaviu Bob, Oana Milas, Anca Suteanu-Simulescu, Mihaela Glavan, Sorin Ursoniu, Lavinia Balint-Marcu, Maria Mogos-Stefan, Silvia Ienciu, Octavian Marius Cretu, Roxana Popescu, Cristina Gluhovschi, Lavinia Iancu, Dragos Catalin Jianu

**Affiliations:** 1 Division of Nephrology, Department of Internal Medicine II, “Victor Babes” University of Medicine and Pharmacy, No. 2, Eftimie Murgu Sq., 300041 Timisoara, Romania; petrica.ligia@umft.ro (L.P.); gadalean.florica@umft.ro (F.G.); bob.flaviu@umft.ro (F.B.); milas.oana@umft.ro (O.M.); anca.simulescu@umft.ro (A.S.-S.); lavinia.balint@umft.ro (L.B.-M.); maria.mogos@umft.ro (M.M.-S.); silvia-ioana.ienciu@umft.ro (S.I.); gluh@umft.ro (C.G.); iuliana.alioani@umft.ro (L.I.); 2 Centre for Molecular Research in Nephrology and Vascular Disease, Faculty of Medicine, “Victor Babes” University of Medicine and Pharmacy, No. 2, Eftimie Murgu Sq., 300041 Timisoara, Romania; vlad.adrian@umft.ro (A.V.); vlad.daliborca@umft.ro (D.V.); dumitrascu.victor@umft.ro (V.D.); tutac.paul@umft.ro (T.P.); sursoniu@umft.ro (S.U.); popescu.roxana@umft.ro (R.P.); jianu.dragos@umft.ro (D.C.J.); 3 Centre for Cognitive Research in Neuropsychiatric Pathology (Neuropsy-Cog), Faculty of Medicine, “Victor Babes” University of Medicine and Pharmacy, No. 2, Eftimie Murgu Sq., 300041 Timisoara, Romania; 4 Center for Translational Research and Systems Medicine, Faculty of Medicine, “Victor Babes” University of Medicine and Pharmacy, No. 2, Eftimie Murgu Sq., 300041 Timisoara, Romania; 5 County Emergency Hospital Timisoara, 300723 Timisoara, Romania; 6 Division of Diabetes, Nutrition, and Metabolic Diseases, Department of Internal Medicine II, “Victor Babes” University of Medicine and Pharmacy, No. 2, Eftimie Murgu Sq., 300041 Timisoara, Romania; 7 Division of Pharmacology, Department of Biochemistry and Pharmacology IV, “Victor Babes” University of Medicine and Pharmacy, No. 2, Eftimie Murgu Sq., 300041 Timisoara, Romania; 8 Division of Public Health and History of Medicine, Department of Functional Sciences III, “Victor Babes” University of Medicine and Pharmacy, No. 2, Eftimie Murgu Sq., 300041 Timisoara, Romania; 9 Division of Surgical Semiology I, Department of Surgery I, “Victor Babes” University of Medicine and Pharmacy, No. 2, Eftimie Murgu Sq., 300041 Timisoara, Romania; 10 Emergency Clinical Municipal Hospital Timisoara, 300041 Timisoara, Romania; 11 Division of Cell and Molecular Biology II, Department of Microscopic Morphology II, “Victor Babes” University of Medicine and Pharmacy, No. 2, Eftimie Murgu Sq., 300041 Timisoara, Romania; 12 Division of Neurology I, Department of Neurosciences VIII, “Victor Babes” University of Medicine and Pharmacy, No. 2, Eftimie Murgu Sq., 300041 Timisoara, Romania

**Keywords:** diabetic kidney disease, vascular endothelial growth factor, placental growth factor, vascular endothelial growth factor receptor 1, kidney, brain

## Abstract

The kidney and the brain share similarities in terms of structure and haemodynamic regime. The aim of the study was to assess a potential correlation of vascular endothelial growth factor (VEGF), soluble vascular endothelial growth factor receptor-1 (sFlt-1), and placental growth factor (PlGF) with biomarkers of podocyte damage, proximal tubular (PT) dysfunction, and endothelial dysfunction, as well as with cerebral vessels haemodynamic indices in neurologic asymptomatic type 2 DM patients. A cohort of 212 patients diagnosed with type 2 DM and 49 age- and gender-matched healthy controls were enrolled in the study. Parameters studied were urinary albumin/creatinine ratio (UACR), biomarkers of podocyte damage (synaptopodin, podocalyxin), PT dysfunction (kidney injury molecule-1-KIM-1, N-acetyl-β-(D)-glucosaminidase-NAG), endothelial dysfunction (P-selectin), VEGF, sFlt-1, and PlGF. The cerebrovascular hemodynamic indices evaluated were intima–media thickness (IMT) in the common carotid arteries (CCAs), the pulsatility index (PI), and the resistivity index (RI) in the internal carotid arteries (ICAs) and middle cerebral arteries (MCAs). Cerebrovascular reactivity (CVR) was assessed by the breath-holding index (BHI). In multivariable regression analysis, serum VEGF correlated directly with UACR, synaptopodin, NAG, serum P-selectin; serum sFlt-1 correlated directly with UACR, synaptopodin, podocalyxin, NAG, KIM-1; serum PlGF correlated negatively with eGFR and directly with UACR, synaptopodin, KIM-1. IMT-CCA correlated indirectly with eGFR and directly with UACR, serum P-selectin, and serum sFlt-1. The PI-ICAs correlated negatively with eGFR and positively with UACR, synaptopodin, serum P-selectin, and serum sFlt-1. The PI-MCAs correlated indirectly with eGFR and directly with synaptopodin, serum P-selectin, and serum sFlt-1. The RI-ICAs had a negative correlation with eGFR and a positive one with UACR, synaptopodin, NAG, KIM-1, urinary sFlt-1, and serum PlGF. The RI-MCAs displayed an indirect correlation with eGFR and a direct correlation with NAG, KIM-1, and serum sFlt-1. The BHT correlated directly with eGFR and negatively with serum P-selectin and serum PlGF. The study shows a significant association of VEGF, sFlt-1, and PlGF with biomarkers of podocyte injury, PT dysfunction, and endothelial dysfunction in early stages of DKD. These pro-angiogenic and anti-angiogenic factors correlated with cerebrovascular haemodynamic indices in neurologic asymptomatic type 2 DM, even in the normoalbuminuric stage of diabetic kidney disease.

## 1. Introduction

Diabetic kidney disease (DKD) represents one of the major vascular complications of both type 1 and type 2 diabetes mellitus (DM) and may be ascribed to over 40% of patients referred to renal replacement therapies worldwide [1]. It is estimated that by 2050, this percentage will reach nearly 50% of all patients with end-stage kidney disease [2]. To date, the trajectories of DKD are well defined, among which the normoalbuminuric phenotype accounts for up to 60% of patients in both types of DM [3,4]. This entity encompasses a urinary albumin/creatinine ratio < 30 mg/g and an eGFR < 60 mL/min/1.73 m^2^. Normoalbuminuric DKD is driven by various mechanisms at the glomerular and tubular levels, which comprise oxidative stress, inflammation, growth factors, genetic, and epigenetic pathways.

Renal microvascularization shares similarities with cerebral vessels in terms of structure and haemodynamic regime. In the course of DKD, there are distinct endothelial territories within the kidney and the brain. Hence, cerebral endothelial dysfunction and cerebral vascular remodeling precede renal endothelial dysfunction, an observation reported in previous studies [5,6].

The vascular endothelial growth factor (VEGF) system has a central role in the initiation and progression of DKD [7]. The VEGF family consists of VEGF-A, the main component, as well as VEGF-B, VEGF-C, VEGF-D, VEGF-E, and placental growth factor (PlGF) [8]. VEGF, which is increased from the early stages of DKD, is implicated as a survival factor for endothelial cells, but also podocytes and the integrity of the glomerular filtration barrier [7]. VEGF is constitutively expressed in podocytes, where it displays both autocrine and paracrine signaling. Also, paracrine signaling of VEGF occurs between podocytes and adjacent endothelial and mesangial cells [9]. Tubular epithelial cells, especially the proximal segment, may produce VEGF, thus supporting the hypothesis that the proximal tubule (PT) could participate actively in intrarenal VEGF synthesis [10]. In addition, VEGF significantly stimulates angiogenesis in the kidney and in the cerebral vessels, as well as in the coronary arteries by forming collateral vessels [7,11]. It has been stated that VEGF can also promote atherosclerosis. Increased serum concentrations of VEGF may be related to atherosclerosis [12]. Controversial reports showed that, in fact, VEGF polymorphism dictates its pro-angiogenic versus atherogenic properties [13]. VEGF is involved in the complex processes of neurovascular development [14] and exerts trophic and protective effects on neurons [15].

Soluble vascular endothelial growth factor receptor-1 (sFlt-1) has a potent and selective VEGF inhibitory action. This soluble Flt-1 is the soluble variant of the VEGF receptor-1, a protein tyrosine kinase with anti-angiogenic properties [9], that binds both VEGF and PlGF [8]. sFlt-1 may also be produced by proximal tubular cells [7]. Placental growth factor potentiates VEGF-mediated angiogenesis and endothelial dysfunction [16]. In addition, PlGF binds to sFlt-1 and impedes its action in diabetic patients, namely albuminuria and lack of vasculogenesis within the kidney and the brain [8].

The aim of the study was to assess a potential correlation between VEGF, sFlt-1, and PlGF and biomarkers of podocyte damage, PT dysfunction, and endothelial dysfunction in the early stages of DKD in patients with type 2 DM. Furthermore, the vascular biomarkers were studied in relation to cerebral vessels’ haemodynamic indices evaluated by neurosonologic methods in order to establish their involvement in atherosclerosis, arteriosclerosis, and collateral vessels formation in neurologic asymptomatic type 2 DM patients.

## 2. Results

### 2.1. Demographic, Clinical, Biological, and Neurosonologic Data of the Patients Studied

The demographic, clinical, biological, and neurosonologic data of the patients and controls are being presented in Table 1 as medians and IQRs, as reported for skewed values of the variables studied (Table 1). Also, in Table 1 are presented the differences with regard to the pro-angiogenic and anti-angiogenic factors and the differences concerning the intima–media thickness in the common carotid arteries (IMT-CCAs), pulsatility index (PI), and resistivity index (RI) in the internal carotid arteries (ICAs) and middle cerebral arteries (MCAs) (PI-ICAs, RI-ICAs, PI-MCAs, and RI-MCAs), which were increased in patients with type 2 DM vs. controls. By contrast, the breath-holding index (BHI) was decreased in patients as compared to healthy control subjects, a fact that reveals a decreased vasodilatory capacity of the cerebral vessels in diabetic patients in response to a vasodilatory stimulus, such as hypercapnia.

### 2.2. Variations in VEGF, sFlt-1, and PlGF Levels in Serum and Urine Correlate with Biomarkers of Podocyte Damage, Proximal Tubule Dysfunction, and Endothelial Dysfunction

Serum and urinary VEGF correlated directly with UACR, urinary synaptopodin, urinary podocalyxin, NGAL, KIM-1, and P-selectin, and negatively with eGFR. Also, the same trend was observed in the univariable regression analysis for serum and urinary sFlt-1 and PlGF across all groups of type 2 DM patients vs. healthy controls (Table 2).

**Figure 1:**
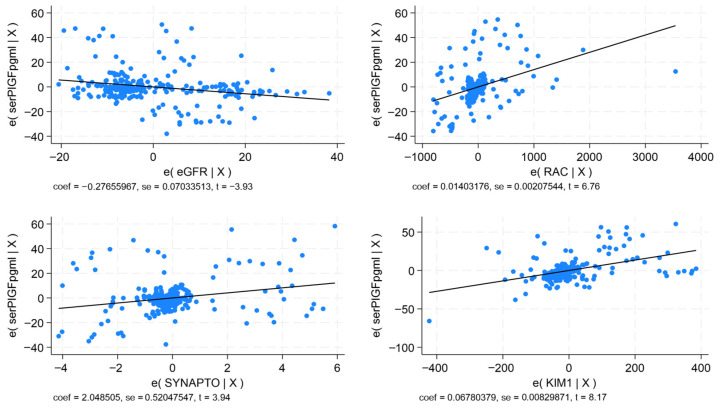
Multivariable regression analysis for serum placental growth factor. Correlations with eGFR, UACR, synaptopodin, and KIM-1.

**Figure 2:**
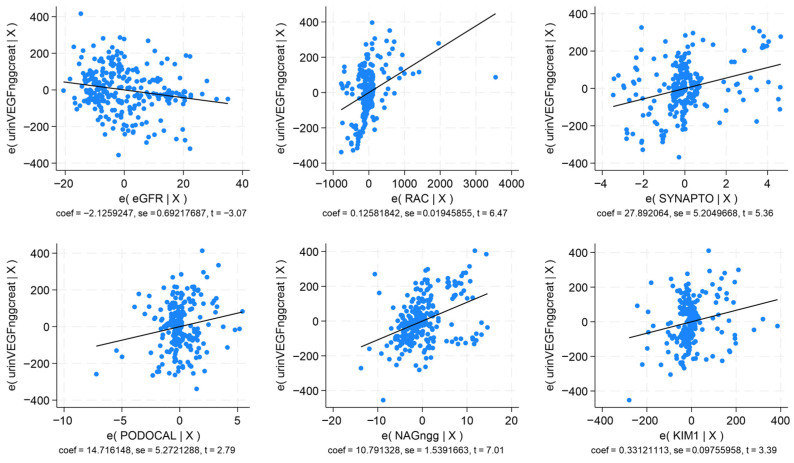
Multivariable regression analysis for urinary vascular endothelial growth factor. Correlations with eGFR, UACR, synaptopodin, pdocalyxin, NAG, and KIM-1.

The significant correlations, as underscored by *p* < 0.0001, demonstrate the intervention of these biomarkers in the progression of DKD. In multivariable regression analysis, VEGF, sFlt-1, and PlGF remained consistently significant in models which show that serum VEGF correlated directly with UACR, synaptopodin, NAG, and serum P-selectin (*p* < 0.0001; R^2^ = 0.785); serum sFlt-1 correlated directly with UACR, synaptopodin, podocalyxin, NAG, and KIM-1 (*p* < 0.0001; R^2^ = 0.769); serum PlGF correlated negatively with eGFR, and directly with UACR, synaptopodin, and KIM-1 (Figure 1; *p* < 0.0001; R^2^ = 0.743). Urinary VEGF correlated negatively with eGFR, and positively with UACR, synaptopodin, podocalyxin, NAG, and KIM-1 (Figure 2; *p* < 0.0001; R^2^ = 0.848).

Urinary sFlt-1 correlated negatively with eGFR, and directly with UACR, synaptopodin, and podocalyxin (*p* < 0.0001; R^2^ = 0.701); urinary PlGF correlated indirectly with eGFR, and directly with UACR, synaptopodin, NAG, and urinary P-selectin (*p* < 0.0001; R^2^ = 0.763) (Table 3). It should be noted that even in normoalbuminuric type 2 DM patients, there is an activation of angiogenic and anti-angiogenic factors, as shown by the increased levels of VEGF, sFlt-1, and PlGF, respectively.

### 2.3. Cerebral Vessels Remodeling Is Impacted by VEGF, sFlt-1, and PlGF in Patients with Type 2 DM and Early DKD

In univariable regression analysis, serum and urinary VEGF correlated directly with IMT-CCA, the RI, and the PI in the ICAs and MCAs, respectively. Also, VEGF showed an indirect correlation with the cerebrovascular reactivity (CVR), measured by the BHI. The levels of sFlt-1 and PlGF paralleled the correlations of VEGF, a fact which shows the concurrent involvement of these angiogenic and anti-angiogenic factors in the atherosclerotic and arteriosclerotic remodeling of cerebral vessels, as well as in potential collateral vessel formation. The cerebrovascular hemodynamic indices also correlated with P-selectin, in conjunction with the angiogenic factors, thus showing the intervention of inflammation in the impact of these factors upon the cerebral vessels (Table 4).

**Figure 3:**
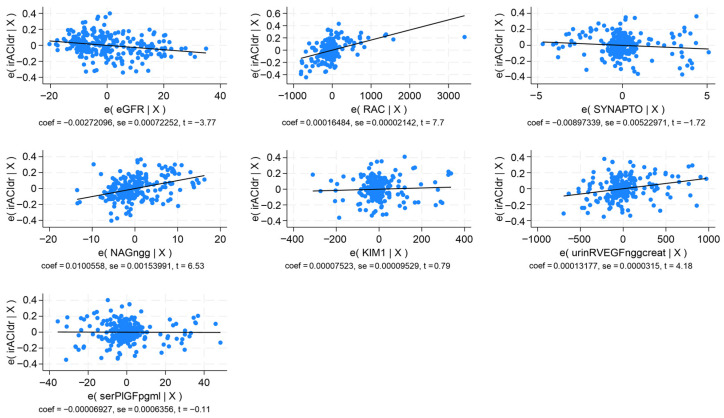
Multivariable regression analysis for the RI in the right internal carotid artery. Correlations with eGFR, UACR, synaptopodin, NAG, KIM-1, VEGF-R1, and PlGF.

**Figure 4:**
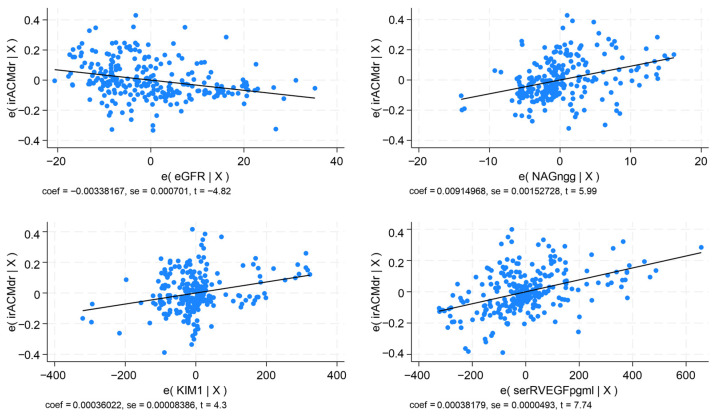
Multivariable regression analysis for the RI in the right middle cerebral artery. Correlations with eGFR, NAG, KIM-1, and VEGF-R1.

In multivariable regression analysis, the models provided revealed that IMT-CCA correlated indirectly with eGFR and directly with UACR, serum P-selectin, and serum sFlt-1 (*p* < 0.0001; R^2^ = 0.716). The PI-ICAs correlated negatively with eGFR and positively with UACR, synaptopodin, serum P-selectin, and serum sFlt-1 (*p* < 0.0001; R^2^ = 0.548). The PI-MCAs showed an indirect correlation with eGFR, and a direct correlation with synaptopodin, serum P-selectin, and serum sFlt-1 (*p* < 0.0001; R^2^ = 0.626). The RI-ICAs had a negative correlation with eGFR and a positive one with UACR, synaptopodin, NAG, KIM-1, urinary sFlt-1, and serum PlGF (Figure 3; *p* < 0.0001; R^2^ = 0.749). The RI-MCAs displayed an indirect correlation with eGFR and a direct one with NAG, KIM-1, and serum sFlt-1 (Figure 4; *p* < 0.0001; R^2^ = 0.799). The BHI correlated directly with eGFR and negatively with serum P-selectin and serum PlGF (*p* < 0.0001; R^2^ = 0.764) (Table 5).

## 3. Discussion

The results of our study show that the pro-angiogenic biomarkers VEGF and PlGF and the anti-angiogenic molecule sFlt-1 are associated with biomarkers of podocyte injury, PT dysfunction, and endothelial dysfunction. Moreover, cerebral vascular remodeling in terms of atherosclerosis and arteriosclerosis is impacted by VEGF, PlGF, and sFlt-1. The novelty of the study resides in the fact that these observations are valid even in normoalbuminuric type 2 DM patients. Thus, the study documented the intervention of pro-angiogenic and anti-angiogenic factors at the renal and cerebrovascular level in the early stages of DKD.

### 3.1. VEGF, PlGF, and sFlt-1 Concentrations Are Implicated Within the Glomeruli and Proximal Tubules in Early DKD of Type 2 DM Patients

Vascular endothelial growth factor has been implicated in the regulation of podocyte functions in an autocrine and paracrine manner [9], also playing a role as a survival factor for endothelial cells [7,17]. Experimental studies performed in diabetic mice provided data that show that the development of proteinuria and glomerular injury is associated with overexpression of VEGF-A in the podocytes. However, increased levels of serum sFlt-1 are also associated with proteinuria and glomerular damage, implying that binding of sFlt-1 to VEGF could preclude its vasoprotective properties [9]. By contrast, tissue-specific inhibition of VEGF by excess sFlt-1 ameliorated albuminuria by possibly blocking the molecular mechanisms of VEGF overexpression at the podocyte and mesangial level [9].

Clinical studies show that normalizing VEGF-A levels with sFlt-1 may ameliorate diabetic nephropathy by reducing podocyte and endothelial damage [18]. In experimental settings, systemic administration of VEGF inhibitors ameliorates proteinuria and glomerular lesions. These contrasting observations suggest that both decreased or increased levels of VEGF may have deleterious effects on the glomerular filtration barrier [19].

In our study, serum and urinary levels of VEGF and sFlt-1 were increased from the normoalbuminuric stage of DKD, similarly to the data reported by Kim N.H. et al., who showed that the urinary VEGF and sFlt-1 levels were significantly higher in the diabetic patients than in the controls [7], even in the normoalbuminuric stage [9]. By contrast to our data, in the study by Ku et al., the plasma VEGF and sFlt-1 levels did not increase and did not differ among the groups studied, possibly due to differences in systemic and local concentration of these factors [9].

The increased levels of VEGF and sFlt-1 in the urine originate mainly from the glomeruli, with a large proportion of VEGF deriving from the podocytes. However, recent research provides data with regard to the tubular origin of VEGF, but also of sFlt-1, the tubule compartment being actively involved in their production, particularly the proximal segment [7,10].

The results of our study show that VEGF and sFlt-1 correlate directly with biomarkers of podocyte damage, PT dysfunction, and endothelial dysfunction across all stages of DKD, thus allowing for the hypothesis that the VEGF/sFlt-1 system could contribute to the progression of diabetic nephropathy by involving complex renal structures.

Placental growth factor is a vasculoprotective factor that potentiates VEGF activities within the glomerulus, but due to its capacity to bind to sFlt-1, PlGF could counteract the VEGF effects [16]. Due to its dual functions, PlGF may act as a protective in vascular repair processes, but also could act as a pro-inflammatory cytokine at the endothelial level [20].

The levels of PlGF in our patients paralleled those of VEGF and exhibited the same correlation with the biomarkers of podocyte injury, PT dysfunction, and endothelial dysfunction. The literature does not provide information concerning the intervention of PlGF at the podocyte and tubular level, but only at the endothelial level, and correlations were only discussed in relation to albuminuria in the course of DKD [21,22,23]. Due to the autocrine and paracrine behavior of the pro-angiogenic factors, we may hypothesize that PlGF could have acted not only upon the endothelial cells, but also upon the podocytes and PT cells, thus inducing expression of biomarkers of tissue-specific damage.

### 3.2. Cerebral Vessels Remodeling and Cerebrovascular Reactivity Are Coordinated by the Expression of VEGF, sFlt-1, and PlGF in Type 2 DM Patients

Vascular endothelial growth factor plays an important role in vascular remodeling, atherosclerosis, and collateral vessel formation [12]. In our study, VEGF correlated with IMT-CCAs, as well as with the RIs and PIs in the ICAs and MCAs, thus showing involvement in important atherosclerotic and arteriosclerotic lesions. In the study by Arenillas et al., VEGF intervention suggested that angiogenesis could be beneficial in patients with symptomatic cerebrovascular disease [24]. However, in our study, the patients included were neurologically asymptomatic, with no stenosis or other vascular lesions on imaging studies. The cerebrovascular remodeling in these patients translated into increased cerebral haemodynamic indices, as well as impaired cerebrovascular reactivity. This latter parameter is closely related to the capacity of small cerebral vessels and collateral vessels to adapt to hypercapnia and hypoxia. The correlations of VEGF and sFlt-1 with the CVR reinforce the hypothesis of the role of angiogenic processes and collateral vessels formation under conditions of metabolic and haemodynamic stress.

Moreover, sFlt-1 levels were positively associated with the impaired haemodynamic parameters, data which is in keeping with the results in the study by Shin et al., who observed that sFlt-1 correlated with carotid artery IMT [25]. Similar data have been reported by Sandhofer et al. in their study, which showed a direct correlation of sFlt-1 with carotid IMT [26].

Placental growth factor levels paralleled those of VEGF and sFlt-1 and displayed similar correlations with the cerebral hemodynamic indices. In the study by Chen Y et al., PlGF was significantly associated with IMT-CCA, the extent of plasma PlGF reflecting subclinical atherosclerosis [20].

Furthermore, in our study, PlGF was associated with a pro-inflammatory cytokine, namely P-selectin, a factor that is signaled in other studies, which show a high content of inflammatory markers in human atherosclerotic plaques [16]. Presumably, the variations in the levels of P-selectin may reflect an adaptive mechanism of the injured endothelium to stabilize plaque integrity in progressive atherosclerotic disease [27,28]. The expression of PlGF is controversial, and it is assumed that low levels are associated with pro-angiogenic effects and plaque stability, while increased levels may display anti-angiogenic effects and plaque instability [8]. In our patients, PIGF also correlated with the CVR even in normoalbuminuric patients. This observation points to a protective/adaptive role of PlGF across all stages of albuminuria by inducing protection to the vascular bed, which presumably resulted from collateral cerebral vessels formation and appropriate microvascular response to hypercapnia. According to the study by Theilade et al., PlGF levels are elevated in relation to undergoing acute or subacute hypoxia. In their study, the authors showed that patients with DM and long-standing normoalbuminuria had higher PlGF levels and were at an unadjusted twofold greater risk of cardiovascular events [22]. In our study, VEGF, sFlt-1, and PlGF increased from the early stages of DKD, but in contrast with other studies, their levels did not decrease with progressive chronic disease [22,29]. Most likely, there are significant differences between systemic and local levels of these factors, with a time-specific pattern imposing variations in their concentrations in relation to the degree of tissue injury.

Our study has several limitations. First, this is a cross-sectional study, which only allows for associations of the parameters studied, but not for a relation of causality of the pro-angiogenic and anti-angiogenic factors with renal and cerebrovascular parameters. Second, although the medication in terms of antihypertensive drugs (ACEIs, ARBs), antidiabetic drugs, and statins was similar in the patients studied, this might represent residual confounders that could bias the interpretation of the data. Third, these biomarkers were assessed in blood and urine, while tissue analysis on kidney biopsies and vascular walls was not performed.

The strength of our study resides in the observation that even in normoalbuminuric type 2 DM patients, pro-angiogenic and anti-angiogenic factors display a particular profile which confers a specific pattern of response at glomerular, proximal tubular, and endothelial level, as well as at cerebrovascular level. Of note, the data derived from our study apply to neurologic asymptomatic type 2 DM patients, in contrast to the vast majority of studies, which included patients with clinically overt cerebrovascular disease.

## 4. Materials and Methods

### 4.1. Cohort/Inclusion/Exclusion Criteria

**Figure 5:**
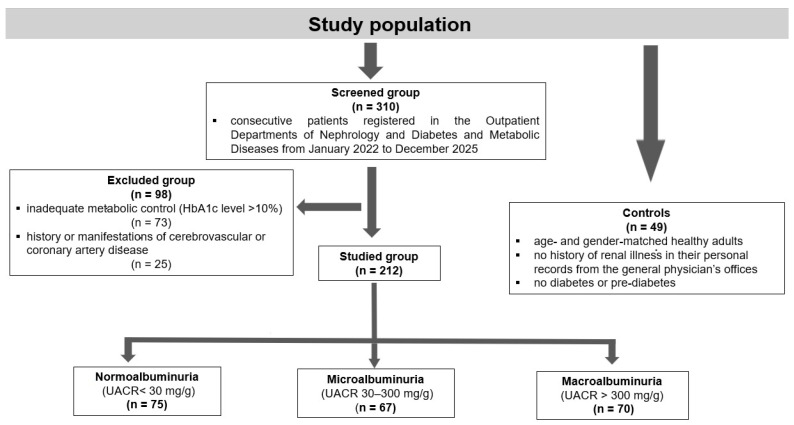
Study population selection and grouping.

A cohort of 212 patients (99 males, 113 females) diagnosed with type 2 DM, selected from a total of 310 consecutive patients registered in the Outpatient Departments of Nephrology and Diabetes and Metabolic Diseases from January 2022 to December 2025, was included in the study. The subjects, aged 50 to 78 years, were evaluated through personal visits and review of their records. The inclusion criteria were duration of DM over 5 years and receiving treatment with oral antidiabetic agents (metformin, gliclazide, SGLT2 inhibitors, or GLP-1 receptor agonists), insulin, angiotensin-converting enzyme inhibitors, angiotensin receptor blockers, and statins. Due to the fact that this study was an exploratory one, the sample size was not calculated. Individuals with inadequate metabolic control (HbA1c level exceeding 10%) or possessing a history or manifestations of cerebrovascular or coronary artery disease, respectively, were excluded from the trial. The 212 patients with type 2 diabetes mellitus were classified into three groups: 75 patients with normoalbuminuria (UACR < 30 mg/g), 67 patients with microalbuminuria (UACR 30–300 mg/g), and 70 patients with macroalbuminuria (UACR > 300 mg/g). A group of 49 age- and gender-matched healthy adults was included, all of whom had no history of renal illness in their personal records from the general physician’s offices, and were screened to exclude diabetes or pre-diabetes, as defined by a HbA1c level of 5.6% or below (Figure 5).

### 4.2. Laboratory Assessments

Serum and urine samples from both patients and controls were stored at −80 °C until analysis. Urinary biomarkers were assessed using the first morning urine sample and expressed as a ratio per urinary creatinine. The biomarkers examined were evaluated utilizing the ELISA technique, as presented previously [5]:

- *Vascular endothelial growth factor* (VEGF, Catalog No. 27171, Immuno-Biological Laboratories Co., Fujioka, Japan), with a sensitivity of less than 7.81 pg/mL, a detection range of 15.63–1000 pg/mL, and an intra-assay coefficient of variance (CV) < 10%.
- *Soluble vascular endothelial growth factor receptor-1* (sFlt-1, Catalog No. abx050237, Abbexa, Cambridge, UK), with a sensitivity of less than 16.5 pg/mL, a detection range of 46.88–3000 pg/mL, and an intra-assay CV < 10%.
- *Placental growth factor* (PlGF, Catalog No. abx250161, Abbexa, Cambridge, UK), with a sensitivity of less than 6.5 pg/mL, a detection range of 15.6–1000 pg/mL, and an intra-assay CV < 10%.
- Podocyte injury biomarkers: *Synaptopodin* (Catalog No. abx055120, Abbexa, Cambridge, UK), with a sensitivity of 0.10 ng/mL, a detection range of 0.156–10 ng/mL, and an intra-assay CV < 10%. *Podocalyxin* (Catalog No. E-EL-H2360, Elabscience, Houston, TX, USA) has a sensitivity of 0.1 ng/mL, a detection range of 0.16–10 ng/mL, and an intra-assay CV < 10%.
- PT dysfunction biomarkers: *Kidney injury molecule-1* (KIM-1, Catalog No. E-EL-H6029, Elabscience, Houston, TX, USA); sensitivity: 4.69 pg/mL; detection range: 7.81–500 pg/mL; intra-assay CV < 10. *N-acetyl-β-(D)-glucosaminidase* (NAG, Catalog No. E-EL-H0898, Elabscience, Houston, TX, USA); sensitivity: 0.94 ng/mL; detection range: 1.56–100 ng/mL; intra-assay CV < 10%.
- Endothelial dysfunction biomarker: *Human SELP* (P selectin) (Catalog No. E-EL-H0917, Elabscience, Houston, TX, USA) with a sensitivity of 0.1 ng/mL, a detection range of 0.16–10 ng/mL, and an intra-assay CV < 10%.

Serum and urine samples were run in triplicate, according to the protocols provided by the manufacturer’s brochure. The eGFR was calculated using the combined serum creatinine–cystatin C (CKD-EPI equation), as recommended in the KDIGO 2024 Guidelines for the Evaluation and Management of CKD [30].

### 4.3. Neurosonologic Ultrasound Methods

Cerebral blood vessels remodeling leads to increased resistance in the examined vessels, which can be evaluated by specific hemodynamic indices, such as the pulsatility index (PI) and the resistance index (RI). The ICAs and the MCAs, which are low-resistance vessels, present with increased PIs and Ris, a fact which is indicative of elevated vascular resistance and decreased vasodilation capacity. This observation points to functional manifestations of vascular remodeling, such as cerebral atherosclerosis, arteriosclerosis, and microangiopathy [31,32,33]. Cerebrovascular reactivity (CVR) represents the increase in blood flow velocity in response to a vasodilatory stimulus [34]. In patients with type 2 DM, CVR is often diminished due to preexisting vasodilation. As a direct consequence, this inadequate response limits the ability of the brain to adapt effectively to changes in cerebral blood flow by eliciting its autoregulatory mechanisms [31,35]. The cerebrovascular hemodynamic indices were assessed by a neurologist blinded to the clinical and biological data of both patients and controls. A high-resolution ultrasound machine (Esaote MyLab 8, Genoa, Italy) equipped with a Color Ultrasound System, featuring two transducers: one with a selectable frequency range of 1.7 to 4 MHz (multifrequency sectorial transducer-phased array) and another with a frequency range of 3.6 to 16 MHz (linear transducer), was utilized for the neurosonologic assessments. The cerebrovascular ultrasound technique was applied as described previously [6].

#### 4.3.1. Carotid Artery Intima–Media Thickness (IMT)

The carotid artery IMT was evaluated bilaterally in the common carotid arteries (CCAs). IMT measures the distance between the luminal–intimal interface and the media-adventitial interface of the carotid arteries. This measurement is displayed in a double-line pattern using ultrasound in brightness mode (B-mode) during a longitudinal view of the carotid artery [36]. Three IMT measurements were applied, and the median value resulting from these measurements was introduced in further analysis. The standard cut-off point for IMT was established at less than 1.0 mm.

#### 4.3.2. Pulsatility Index (PI) and Resistivity Index (RI)

The PI and the RI were evaluated bilaterally in the ICAs using continuous-wave Doppler ultrasound at a frequency of 4 MHz. In addition, the MCAs were assessed with pulsed-wave Doppler ultrasound at a frequency of 2 MHz. Gosling’s PI, formulated as (systolic flow velocity–diastolic flow velocity)/mean flow velocity (with a standard value of <1), and Pourcelot’s RI, calculated as (systolic flow velocity–diastolic flow velocity)/systolic flow velocity (with a standard value of <0.7), were calculated automatically using the above-mentioned formulas [32].

#### 4.3.3. Cerebrovascular Reactivity

The CVR, which measures the responsiveness of cerebral blood vessels to vasodilation stimuli, was evaluated using the transcranial Doppler breath-holding test (BHT) in the MCAs bilaterally. The procedure began after the participants breathed room air for approximately 4 min, followed by a breath-holding phase lasting 30 s at the end of a normal expiration. The complex hemodynamic parameters, such as mean flow velocity (MFV), systolic flow velocity, and diastolic flow velocity, were assessed at rest, during the breath-holding maneuver, and by the end of the BHT, at the peak of hypercapnia, which served as the vasodilatory stimulus. The maneuver was repeated after a 2–3 min rest period, in order to allow the MFV to return to baseline levels. The mean MFV recorded in the MCAs and the mean breath-holding index (BHI) were then calculated. The BHI is defined as the percentage increase in MFV in the MCAs during the breath-holding maneuver, divided by the duration of breath holding in seconds. It is expressed with the formula [(Vbh − Vr/Vr) × 100 s^−1^], where Vbh is the MFV in the MCAs at the end of breath holding, Vr is the MFV at rest, and s^−1^ indicates per second of breath holding. The standard value for the BHI is 1.2 ± 0.6 [35].

### 4.4. Statistical Analysis

The statistical methods were applied in accordance with the requirements of case-series exploratory studies. Clinical and biological data are presented as medians and interquartile ranges (IQRs) for variables that exhibit a skewed distribution. Differences between subgroups were analyzed using the Mann–Whitney U test for comparisons between two groups and the Kruskal–Wallis test for comparisons among four groups based on the distribution of values. Regression analyses were performed to assess the significance of the relationships between cerebrovascular hemodynamic indices and serum and urinary levels of VEGF, sFlt-1, and PlGF, as well as other continuous variables, including synaptopodin, podocalyxin, KIM-1, NAG, P-selectin, UACR, and the eGFR. A univariable regression analysis was conducted to evaluate the significance of the relationships among continuous variables across all four groups, which included pooled data from normal, microalbuminuric, macroalbuminuric patients, and healthy controls, respectively. Only those variables that showed significance in the univariable regression analysis were included in the subsequent multivariable regression analysis models. The threshold for statistical significance was set at *p* < 0.05, and this study was conducted using Stata 19 (StataCorp, College Station, TX, USA). 

## 5. Conclusions

The data provided by our study conducted on type 2 DM patients shows a significant association of VEGF, sFlt-1, and PlGF with biomarkers of podocyte injury, PT dysfunction, as well as of endothelial dysfunction in early stages of DKD.

Furthermore, these pro-angiogenic and anti-angiogenic factors correlated with cerebrovascular haemodynamic indices in neurologic asymptomatic type 2 DM, even in the normoalbuminuric stage of DKD. The relation of these factors with the kidney and the brain, which have in common structural and functional traits, is of clinical importance due to the fact that the results obtained may be translated into practice in normoalbuminuric patients with type 2 DM, often perceived as unaffected by diabetic complications and neglected in clinical trials. The dissociated behavior of the pro-angiogenic and anti-angiogenic factors within the two microvascular territories may derive from a tissue-specific response among the segments of the nephron, as well as in the cerebral vasculature.

Further studies, on larger cohorts, in a multi-center approach, would be beneficial in patients with type 2 DM in the early stages of DKD.

## Figures and Tables

**Table 1:** Demographic, clinical, and biological data of patients with type 2 DM and healthy controls.

Parameter	Healthy Controls (N = 49, 18.8%)	Normoalbuminuric Patients (N = 75, 28.7%)	Microalbuminuric Patients (N = 67, 28.7%)	Macroalbuminuric Patients (N = 70, 26.8%)	Total (N = 261, 100%)	*p*-Value
**Age (years)**	68 (65–70)	69 (65–72)	70 (66–75)	69 (66–74)	69 (66–73)	0.067
**Serum creatinine (mg/dL)**	0.8 (0.7–0.8) ^#,♦,◊,^*	1.18 (1.1–1.3)	1.20 (1.1–1.3) ^■^	1.26 (1.2–1.5)	1.17 (1–1.3)	<0.0001
**eGFR (mL/min/1.73 m^2^)**	83 (80.6–87.7) ^#,♦,◊,^*	57.2 (55.6–58.6) ^⁑,○^	54.7 (52.1–58.8) ^■^	51 (45–57)	57 (52.2–62.3)	<0.0001
**HbA1c (%)**	5 (4.8–5.2) ^#,♦,◊,^*	7 (6.5–7.9) ^♣,○^	8.2 (7.5–9.6) ^■^	8.2 (7.9–9)	7.7 (6.5–8.4)	<0.0001
**Cholesterol (mg/dL)**	122 (112–150) ^#,♦,◊,^*	160 (131–202) ^○^	167 (131–201) ^□^	199.5 (154–223)	164 (131–202)	<0.0001
**Triglycerides (mg/dL)**	97 (88–100) ^#,♦,◊,^*	145 (105–179) ^○^	150 (119–215) ^●^	183 (145–255)	145 (102–203)	<0.0001
**UACR (mg/g)**	11.4 (10.2–17.7) ^#,♦,◊,^*	23.1 (15.2–26.9) ^♣,○^	88.9 (61.5–131.1) ^■^	648.4 (450–1303.9)	50.9 (18.2–311.9)	<0.0001
**Synaptopodin/creat (ng/g)**	0.8 (0.6–0.9) ^#,♦,◊,^*	1.8 (1.3–1.9) ^♣,○^	2.4 (2.2–2.6) ^■^	5.3 (3.1–8.7)	2.1 (1.57–2.9)	<0.0001
**Podocalyxin/creat (ng/g)**	0.3 (0.3–0.6) ^#,♦,◊,^*	1.2 (1–1.3) ^♣,○^	3.5 (3.3–4.3) ^■^	8.2 (6.9–9.2)	2.9 (1–4.6)	<0.0001
**NAG/creat (ng/g)**	2.6 (2.3–2.9) ^#,♦,◊,^*	4.9 (3.1–10.2) ^♣,○^	11.2 (10.4–17.9) ^■^	17.8 (16.9–22.5)	10.4 (3.4–17.3)	<0.0001
**KIM-1/creat (pg/g)**	45 (40.3–47.4) ^#,♦,◊,^*	79.5 (66.9–94.6) ^♣,○^	141.3 (130.3–149.1) ^■^	415.1 (311.6–484.7)	121.9 (65.7–175)	<0.0001
**Serum P-selectin (ng/mL)**	0.5 (0.5–0.7) ^#,♦,◊,^*	1.3 (1.2–1.5) ^♣,○^	4.6 (4.2–4.6) ^■^	8.4 (7.1–9.2)	5.6 (1.2–6.4)	<0.0001
**Urinary P-selectin/creat (ng/g)**	0.3 (0.3–0.4) ^#,♦,◊,^*	1 (0.8–1.3) ^♣,○^	3.4 (3.1–3.6) ^■^	6.9 (5.8–7.7)	2.5 (0.8–4.8)	<0.0001
**Serum sFlt-1 (pg/mL)**	64.1 (57–81.3) ^#,♦,◊,^*	174.2 (116.9–185.8) ^♣,○^	263.2 (210.9–448.9) ^■^	664.4 (580.6–930.6)	209.1 (116.5–484.7)	<0.0001
**Urinary sFlt-1/creat (pg/g)**	71.9 (65.5–98.1) ^#,♦,◊,^*	261.7 (163.9–185.8) ^♣,○^	329 (309.2–561.2) ^■^	924.2 (886.3–1661.9)	300.2 (160.9–788.5)	
**Serum VEGF (pg/mL)**	54.4 (49.1–69.1) ^#,♦,◊,^*	110.7 (87.7–141.5) ^♣,○^	237.2 (217.3–277.4) ^■^	566.5 (452.8–746.7)	187.6 (91.3–341)	<0.0001
**Urinary VEGF/creat (pg/g)**	70.7 (59.2–84.1) ^#,♦,◊,^*	163.4 (123.3–233.4) ^♣,○^	416.3 (278.7–518.3) ^■^	841.9 (732.7–971.9)	277.5 (136.6–557.4)	<0.0001
**Serum PlGF (pg/mL)**	9.5 (8.9–13.1) ^#,♦,◊,^*	17.6 (14.9–19.7) ^♣,○^	25.7 (22.1–27) ^■^	67.3 (48.9–88.3)	21.4 (15.5–134.7)	<0.0001
**Urinary PIGF/creat (pg/g)**	12.4 (8.9–13.1) ^#,♦,◊,^*	37.1 (34.2–40.1) ^♣,○^	51.6 (41.8–55.6) ^■^	111 (79.9–144.1)	41.9 (34–63.4)	<0.0001
**IMT-rCCA**	0.7 (0.6–0.7) ^#,♦,◊,^*	0.8 (0.8–0.9) ^♣,○^	0.9 (0.9–1.1) ^■^	1.2 (1.1–1.4)	0.9 (0.8–1.1)	<0.0001
**PI-rICA**	0.8 (0.7–0.9) ^#,♦,◊,^*	0.9 (0.8–1) ^♣,○^	1 (0.9–1.2) ^■^	1.3 (1.1–1.3)	0.9 (0.9–1.2)	<0.0001
**PI-rMCA**	0.6 (0.6–0.7) ^#,♦,◊,^*	0.8 (0.7–0.9) ^♣,○^	0.9 (0.8–1.1) ^■^	1.2 (0.9–1.2)	0.9 (0.7–1.1)	<0.0001
**RI-rICA**	0.6 (0.5–0.6) ^#,♦,◊,^*	0.7 (0.7–0.8) ^♣,○^	0.9 (0.8–1) ^■^	1.2 (1.1–1.3)	0.9 (0.6–1.1)	<0.0001
**RI-rMCA**	0.5 (0.5–0.6) ^#,♦,◊,^*	0.7 (0.6–0.7) ^♣,○^	1 (0.9–1) ^■^	1.2 (1.1–1.3)	0.9 (0.6–1.1)	<0.0001
**BHI**	1.1 (1.1–1.2) ^#,♦,◊,^*	0.8 (0.7–0.9) ^♣,○^	0.5 (0.5–0.6) ^■^	0.4 (0.4–0.5)	0.6 (0.5–0.9)	<0.0001

Clinical and biological data are presented as medians and IQR for variables with skewed distribution. Significance between healthy controls and normoalbuminuric group, ^#^ *p* < 0.001; significance between healthy controls and microalbuminuric group, ^♦^ *p* < 0.001; significance between healthy controls and macroalbuminuric group, ^◊^ *p* < 0.001; significance between normoalbuminuric group and microalbuminuric group ^♣^ *p* < 0.001, ^⁑^ *p* = 0.007; significance between normoalbuminuric group and macroalbuminuric group ^●^ *p* < 0.001; significance between microalbuminuric group and macroalbuminuric group; ^■^ *p* < 0.001; ^○^ *p* = 0.008; ^□^ *p* = 0.001; significance between healthy controls vs. normoalbuminuric group vs. microalbuminuric group vs. macroalbuminuric group, * *p* < 0.001; eGFR: estimated glomerular filtration rate; UACR: urinary albumin/creatinine ratio; KIM-1/creat: urinary kidney injury molecule-1/creatinine ratio; NAG/creat: N-acetyl-β-(D)-glucosaminidase/creatinine ratio; podocalyxin/creat: podocalyxin/creatinine ratio; synaptopodin/creat: synaptopodin/creatinine ratio; HbA1C: glycated hemoglobin; sFlt-1: soluble fms-like tyrosine kinase-1, VEGF: vascular endothelial growth factor; PlGF: placental growth factor; IMT-rCCA: intima–media thickness right common carotid artery; PI-rICA: pulsatility index right internal carotid artery; PI-rMCA: pulsatility index right middle cerebral artery; RI-rICA: resistivity index right internal carotid artery; RI-rMCA: resistivity index right middle cerebral artery; BHI: breath-holding index.

**Table 2:** Univariable regression analysis for VEGF, sFlt-1, and PlGF.

Parameter	Variable	R^2^	Coef. β	*p*-Value
**Serum VEGF**	eRFG	0.296	−9.443	<0.001
	UACR	0.499	0.306	<0.001
	Synaptopodin	0.591	77.116	<0.001
	Serum P-selectin	0.679	62.853	<0.001
	Podocalyxin	0.614	55.029	<0.001
	KIM-1	0.603	1.183	<0.001
	NAG	0.562	22.915	<0.001
**Urinary VEGF**	eRFG	0.333	−13.142	<0.001
	UACR	0.530	0.414	<0.001
	Synaptopodin	0.609	102.879	<0.001
	Podocalyxin	0.668	80.793	<0.001
	NAG	0.626	31.731	<0.001
	KIM-1	0.647	1.608	<0.001
	Urinary P-selectin	0.707	99.359	<0.001
**Serum sFlt-1**	eRFG	0.282	−11.181	<0.001
	UACR	0.504	0.373	<0.001
	Synaptopodin	0.555	90.768	<0.001
	Podocalyxin	0.606	71.182	<0.001
	NAG	0.521	26.771	<0.001
	KIM-1	0.634	1.472	<0.001
	Serum P-selectin	0.642	74.144	<0.001
**Urinary sFlt-1**	eRFG	0.273	−18.557	<0.001
	UACR	0.394	0.556	<0.001
	Synaptopodin	0.497	144.845	<0.001
	Podocalyxin	0.642	123.586	<0.001
	NAG	0.444	41.675	<0.001
	KIM-1	0.636	2.486	<0.001
	Serum P-selectin	0.640	147.528	<0.001
**Serum PIGF**	eRFG	0.284	−1.018	<0.001
	UACR	0.521	0.034	<0.001
	Synaptopodin	0.509	7.886	<0.001
	Podocalyxin	0.590	6.369	<0.001
	NAG	0.471	2.308	<0.001
	KIM-1	0.631	0.133	<0.001
	Serum P-selectin	0.651	6.763	<0.001
**Urinary PIGF**	eRFG	0.327	−1.763	<0.001
	UACR	0.502	0.544	<0.001
	Synaptopodin	0.559	13.310	<0.001
	Podocalyxin	0.546	9.865	<0.001
	NAG	0.549	4.017	<0.001
	KIM-1	0.545	0.199	<0.001
	Serum P-selectin	0.608	12.446	<0.001

UACR: urinary albumin/creatinine ratio; eGFR: estimated glomerular filtration rate; KIM-1: urinary kidney injury molecule-1; NAG: N-acetyl-β-(D)-glucosaminidase; sFlt-1: soluble fms-like tyrosine kinase-1; VEGF: vascular endothelial growth factor; PlGF: placental growth factor.

**Table 3:** Multivariable regression analysis for VEGF, sFlt-1, and PlGF.

Parameter	Variable	Coef. β	*p*-Value	95% CI	Prob. > F	R^2^
**Serum VEGF**	UACR	0.10238	<0.0001	0.06914 to 0.1356	0.000	0.785
	Synaptopodin	23.4798		14.2392 to 32.7204	0.000	
	NAG	6.5047		3.7015 to 9.3078	0.000	
	Serum P-selectin	25.797		17.3681 to 34.225	0.000	
**Serum sFlt-1**	UACR	0.121	<0.0001	0.0772 to 0.1643	0.000	0.769
	Synaptopodin	23.145		11.483 to 34.808	0.000	
	Podocalyxin	11.293		−0.454 to −23.041	0.059	
	NAG	7.624		4.320 to 10.928	0.000	
	KIM-1	0.512		0.29403 to 0.73002	0.000	
**Serum PIGF**	eGFR	−0.28	<0.0001	−0.4151 to −0.1381	0.000	0.7432
	UACR	0.0149		0.0099 to 0.01811	0.000	
	Synaptopodin	2.049		1.0235 to 3.0735	0.000	
	KIM 1	0.068		0.0515 to 0.0841	0.000	
**Urinary VEGF**	eGFR	−2.126	<0.0001	−3.489 to −0.7628	0.002	0.848
	UACR	0.126		0.0875 to 0.1641	0.000	
	Synaptopodin	27.892		17.642 to 38.1425	0.000	
	Podocalyxin	14.716		4.333 to 25.099	0.006	
	NAG	10.791		7.7602 to 13.8225	0.000	
	KIM	0.331		0.1391 to 0.52334	0.001	
**Urinary sFlt-1**	eGFR	−3.949	<0.0001	−6.781 to −1.1185	0.006	0.702
	UACR	0.173		0.0951 to 0.2518	0.000	
	Synaptopodin	30.833		8.6673 to 52.9997	0.007	
	Podocalyxin	79.962		63.1196 to 96.8036	0.000	
**Urinary PIGF**	eGFR	−0.437	<0.0001	−0.6687 to −0.2062	0.000	0.763
	UACR	0.021		0.145 to 0.027	0.000	
	Synaptopodin	4.374		2.648 to 6.099	0.000	
	NAG	1.220		0.695 to 1.746	0.000	
	Urinary P selectin	2.856		1.003 to 4.709	0.003	

UACR: urinary albumin/creatinine ratio; eGFR: estimated glomerular filtration rate; KIM-1: urinary kidney injury molecule-1; NAG: N-acetyl-β-(D)-glucosaminidase; sFlt-1: soluble fms-like tyrosine kinase-1; VEGF: vascular endothelial growth factor; PlGF: placental growth factor.

**Table 4:** Univariable regression analysis for cerebral hemodynamic indices.

Parameter	Variable	R^2^	Coef. β	*p*-Value
**IMT-rCCA**	eGFR	0.412	−0.011	
	UACR	0.356	0.0002	<0.001
	Synaptopodin	0.365	0.611	<0.001
	Podocalyxin	0.555	0.057	<0.001
	NAG	0.407	0.019	<0.001
	KIM-1	0.569	0.001	<0.001
	Urinary P-selectin	0.608	0.071	
	Serum P-selectin	0.628	0.061	<0.001
	Serum VEGF	0.493	0.0007	<0.001
	Urinary VEGF	0.562	0.0005	<0.001
	Serum sFlt-1	0.582	0.0006	
	Urinary sFlt-1	0.565	0.0004	
	Serum PIGF	0.472	0.006	
	Urinary PIGF	0.463	0.004	
**PI-rICA**	eGFR	0.271	−0.008	<0.001
	UACR	0.296	0.0002	<0.001
	Synaptopodin	0.248	0.046	<0.001
	Podocalyxin	0.412	0.044	<0.001
	NAG	0.336	0.162	<0.001
	KIM-1	0.391	0.0009	<0.001
	Urinary P-selectin	0.446	0.055	<0.001
	Serum P-selectin	0.467	0.048	<0.001
	Serum VEGF	0.408	0.0006	<0.001
	Urinary VEGF	0.463	0.0005	
	Serum sFlt-1	0.458	0.0005	
	Urinary sFlt-1	0.422	0.0003	
	Serum PIGF	0.375	0.005	
	Urinary PIGF	0.380	0.003	
**PI-rMCA**	eGFR	0.389	−0.010	<0.001
	UACR	0.301	0.0002	<0.001
	Synaptopodin	0.275	0.049	<0.001
	Podocalyxin	0.462	0.049	<0.001
	NAG	0.409	0.019	<0.001
	KIM-1	0.426	0.0009	<0.001
	Urinary P-selectin	0.499	0.060	<0.001
	Serum P-selectin	0.521	0.052	<0.001
	Serum VEGF	0.434	0.0006	<0.001
	Urinary VEGF	0.501	0.0005	
	Serum sFlt-1	0.496	0.0006	
	Urinary sFlt-1	0.457	0.0003	
	Serum PIGF	0.402	0.005	
	Urinary PIGF	0.421	0.003	
**RI-rICA**	eGFR	0.332	−0.010	<0.001
	UACR	0.524	0.0003	<0.001
	Synaptopodin	0.367	0.064	<0.001
	Podocalyxin	0.527	0.574	<0.001
	NAG	0.553	0.239	<0.001
	KIM-1	0.488	0.001	<0.001
	Urinary P-selectin	0.603	0.734	<0.001
	Serum P-selectin	0.621	0.631	<0.001
	Serum VEGF	0.588	0.0008	<0.001
	Urinary VEGF	0.663	0.0007	<0.001
	Serum sFlt-1	0.619	0.0007	<0.001
	Urinary sFlt-1	0.552	0.0004	<0.001
	Serum PIGF	0.538	0.007	<0.001
	Urinary PIGF	0.572	0.045	<0.001
**RI-MCA**	eGFR	0.394	−0.013	<0.001
	UACR	0.359	0.003	<0.001
	Synaptopodin	0.471	0.079	<0.001
	Podocalyxin	0.706	0.073	<0.001
	NAG	0.602	0.027	<0.001
	KIM-1	0.603	0.001	<0.001
	Urinary P-selectin	0.763	0.091	<0.001
	Serum P-selectin	0.794	0.079	<0.001
	Serum VEGF	0.635	0.0009	<0.001
	Urinary VEGF	0.728	0.0007	<0.001
	Serum sFlt-1	0.702	0.0008	<0.001
	Urinary sFlt-1	0.662	0.0005	<0.001
	Serum PIGF	0.570	0.008	<0.001
	Urinary PIGF	0.571	0.005	<0.001
**BHI**	eGFR	0.549	0.015	<0.001
	UACR	0.245	−0.0002	<0.001
	Synaptopodin	0.369	−0.709	<0.001
	Podocalyxin	0.575	−0.066	<0.001
	NAG	0.540	−0.026	<0.001
	KIM-1	0.458	−0.001	<0.001
	Urinary P-selectin	0.623	−0.083	<0.001
	Serum P-selectin	0.648	−0.071	<0.001
	Serum VEGF	0.496	−0.0008	<0.001
	Urinary VEGF	0.581	−0.007	<0.001
	Serum sFlt-1	0.531	−0.007	<0.001
	Urinary sFlt-1	0.486	−0.0004	<0.001
	Serum PIGF	0.426	−0.007	<0.001
	Urinary PIGF	0.481	−0.005	<0.001

UACR: urinary albumin/creatinine ratio; eGFR: estimated glomerular filtration rate; KIM-1: urinary kidney injury molecule-1; NAG: N-acetyl-β-(D)-glucosaminidase; IMT-rCCA: intima–media thickness right common carotid artery; PI-rICA: pulsatility index right internal carotid artery; PI-rMCA: pulsatility index right middle cerebral artery; RI-rICA: resistivity index right internal carotid artery; RI-rMCA: resistivity index right middle cerebral artery; BHI: breath-holding index, sFlt-1: soluble fms-like tyrosine kinase-1, VEGF: vascular endothelial growth factor; PlGF: placental growth factor.

**Table 5:** Multivariable regression analysis for cerebral hemodynamic indices.

Parameter	Variable	Coef. β	*p*-Value	95% CI	Prob. > F	R^2^
**IMT-rCCA**	eGFR	−0.0043	<0.0001	−0.00575 to −0.00291	0.000	0.716
	UACR	0.00003		−3.55 to 0.00007	0.000	
	Serum P-selectin	0.0299		0.02106 to 0.0388	0.000	
	sFlt-1	0.00029		0.00011 to 0.000318	0.000	
**PI-ICA**	eGFR	−0.0023	<0.0001	−0.00399 to −0.00071	0.000	0.549
	UACR	0.00005		6.65 to 0.0001	0.000	
	Synaptopodin	0.0184		0.0311 to 0.00563	0.000	
	Serum P-selectin	0.0283		0.01721 to 0.03941	0.000	
	sFlt-1	0.00025		0.00012 to 0.00037	0.000	
**PI-MCA**	eGFR	−0.0045	<0.0001	−0.00599 to −0.00291	0.000	0.626
	Synaptopodin	−0.0157		−0.02704 to −0.0035	0.000	
	Serum P-selectin	0.0287		0.0182 to 0.03916	0.000	
	Serum sFlt-1	0.0002		0.00017 to 0.00039	0.000	
**RI-rICA**	eGFR	−0.0027	<0.0001	−0.00414 to −0.0013	0.000	0.749
	UACR	0.0002		0.000123 to 0.00021	0.000	
	Synaptopodin	0.0089		0.019273 to 0.00133	0.000	
	NAG	0.0101		0.00702 to 0.0131	0.000	
	KIM-1	0.00007		0.00011 to 0.00026	0.000	
	Urinary sFlt-1	0.0001		0.000069 to 0.000194	0.000	
	Serum PIGF	0.00006		0.00132 to 0.001183	0.000	
**RI-rMCA**	eGFR	−0.0034	<0.0001	−0.0032 to −0.00031	0.000	0.799
	NAG	0.0091		0.00008 to 0.00062	0.000	
	KIM-1	0.0004		0.00098 to 0.0078	0.000	
	Serum sFlt-1	0.0004		0.0145 to 0.0394	0.000	
**BHI**	eGFR	0.0085	<0.0001	0.0069 to 0.0099	0.000	0.764
	Serum P-selectin	−0.055		−0.0641 to −0.0454	0.000	
	Serum PIGF	−0.0007		−0.00034 to −0.00181	0.001	

UACR: urinary albumin/creatinine ratio; eGFR: estimated glomerular filtration rate; KIM-1: urinary kidney injury molecule-1; NAG: N-acetyl-β-(D)-glucosaminidase; sFlt-1: soluble fms-like tyrosine kinase-1, VEGF: vascular endothelial growth factor; PlGF: placental growth factor; IMT-rCCA: intima–media thickness right common carotid artery; PI-rICA: pulsatility index right internal carotid artery; PI-rMCA: pulsatility index right middle cerebral artery; RI-rICA: resistivity index right internal carotid artery; RI-rMCA: resistivity index right middle cerebral artery; BHI: breath-holding index.

## Data Availability

The data that support the findings of this study are available from the corresponding author upon reasonable request.
